# SURGICAL TREATMENT OF GASTRIC STUMP CANCER: A COHORT STUDY OF 51 PATIENTS

**DOI:** 10.1590/0102-6720202400056e1850

**Published:** 2025-01-13

**Authors:** Eric DRIZLIONOKS, Valdir TERCIOTI, João de Souza COELHO, Nelson Adami ANDREOLLO, Luiz Roberto LOPES

**Affiliations:** 1 Universidade Estadual de Campinas, Faculty of Medical Sciences, Department of Surgery, Digestive Diseases Surgical Unit – Campinas (SP), Brazil.

**Keywords:** Gastric stump, Stomach neoplasms, Gastrectomy, Postoperative complications, Survival, Coto gástrico, Neoplasias gástricas, Gastrectomia, Complicações pós-operatórias, Sobrevida

## Abstract

**BACKGROUND:**

Gastric stump neoplasia is defined as a neoplasia that arises in the gastric remnant after at least 5 years of interval from the first gastric resection.

**AIMS:**

The aim of this study was to analyze 51 patients who underwent total and subtotal gastrectomy and multi-visceral resections in patients with gastric stump cancer.

**METHODS:**

The hospital records of 51 patients surgically treated for gastric stump cancer between 1989 and 2019 were reviewed. The following data were analyzed: sex, age group, the interval between the first surgery and the diagnosis of gastric stump cancer, location of the ulcer that motivated the gastrectomy, type of reconstruction, tumor resectability, surgery performed, reconstruction of the digestive tract, associated surgical procedures, postoperative complications using the Clavien-Dindo classification, disease staging, and survival.

**RESULTS:**

There were 43 (83.3%) men, with a mean age of 66.9 years. The mean interval between the initial gastrectomy and surgery for the treatment of gastric stump neoplasia was 34.7 years. All had previously undergone Billroth II reconstruction. Most patients underwent total gastrectomy (35 cases – 68.6%), followed by subtotal gastrectomy (6 cases – 11.8%), and the remainder were considered unresectable (10 patients – 19.6%), undergoing jejunostomy for nutritional support. Multi-visceral resections consisted of splenectomies, cholecystectomies, hepatectomies, partial colectomies, pancreatectomies, enterectomies, and nephrectomies. Among the patients who had the lesion resected, the mean follow-up time was 34.2 months (standard deviation: 47.6 months), the overall survival at 3 years was 43.6%, and the survival at 5 years was 29.7%.

**CONCLUSION:**

The treatment of gastric stump neoplasia is still challenging and difficult, and personalized follow-up strategies should be focused on high-risk patients, offering opportunities for early intervention, better clinical outcomes, and long-term survival.

## RESUMO

**RACIONAL:**

A neoplasia de coto gástrico é definida como uma neoplasia que surge no remanescente gástrico após pelo menos 5 anos de intervalo da primeira ressecção gástrica.

**OBJETIVOS:**

Analisar 51 pacientes submetidos a gastrectomia total, subtotal e ressecções multiviscerais em doente portadores de câncer do coto gástrico.

**MÉTODOS:**

Revisados os arquivo hospitalares de 51 pacientes tratados cirùrgicamente por cancer do coto gástrico entre 1989 e 2019. Foram analisados o sexo, o grupo etário, o intervalo entre a primeira cirurgia e o diagnóstico do câncer de coto gástrico, o local da úlcera que motivou a gastrectomia, o tipo de reconstrução, a ressecabilidade do tumor, a cirurgia realizada, a reconstrução do trânsito digestivo, procedimentos cirúrgicos associados, as complicações pós-operatórias empregando a classificação de Clavien-Dindo, o estadiamento da doença e a sobrevida.

**RESULTADOS:**

Foram 43 (83,3%) homens, com uma média de idade de 66,9 anos. O intervalo médio entre a gastrectomia inicial e a cirurgia para o tratamento da neoplasia do coto gástrico foi de 34,7 anos. Todos haviam sido prèviamente submetidos à reconstrução a Billroth II. A maioria dos doentes foi submetida a gastrectomia total (35 casos – 68,6%), seguido por uma gastrectomia subtotal (6 casos – 11,8%) e os demais foram considerados irrecáveis (10 pacientes – 19,6%), sendo submetidos a jejunostomia, para suporte nutricional. Resseções multiviscerais consistiram de esplenectomias, colecistectomias, hepatectomias, colectomias parciais, pancreatectomias, enterectomias e nefrectomia. Entre os doentes que tiveram a lesão ressecada, a média do tempo de seguimento foi 34,2 meses (desvio padrão: 47,6 meses), e a sobrevida global em 3 anos foi de 43,6% e a sobrevida em 5 anos foi de 29,7%.

**CONCLUSÃO:**

O tratamento da neoplasia de coto gástrico ainda é desafiador, difícil e estratégias de acompanhamento personalizadas, devendo ser focadas em pacientes de alto risco, oferecendo oportunidade para uma intervenção precoce, melhores desfechos clínicos e sobrevida a longo prazo.

## INTRODUCTION

Gastric stump neoplasia, also known as gastric remnant cancer, was initially described in 1922 by Balfour, who observed neoplastic lesions in patients undergoing partial gastrectomies between 5 and 8 years after the initial surgery^2^. In 1938, Prinz established specific criteria to distinguish gastric stump neoplasia from conventional gastric cancer, stipulating that the initial gastric resection should be due to a benign condition and that the manifestation of carcinoma should occur at least 5 years after the first surgery^13^.

Later, the concept of gastric stump cancer has been expanded to include tumors that develop in the gastric remnant following surgeries for primary gastric neoplasias. These tumors, which typically occur about 10 years after the first surgery, are considered second primary tumor^10,14,17^.

Several factors are identified as carcinogenic risk factors for gastric stump neoplasia, among which the following stand out: decreased serum gastrin levels, hypochlorhydria, the action of nitrites, nitrates, and N-nitroso compounds, biliary reflux frequently associated with Billroth II reconstruction, *Helicobacter pylori* infection, Epstein-Barr virus infection, and the presence of lymph nodes affected by the initially treated tumor^1,14,16,18^.

Historically, the introduction of H_2_ receptor antagonists in the 1970s for the treatment of peptic ulcers and later proton pump inhibitors had a significant impact on reducing peptic ulcer complications and the need for gastric surgeries, which possibly contributed to the decrease in the incidence of conventional gastric cancer, including gastric stump neoplasia^6,10,22^. Despite the lack of specific data on the epidemiology of gastric stump neoplasia in Brazil, it is plausible that the prevalence has been affected by the accessible use of proton pump inhibitors, following the global pattern^6,22^.

The objective of this study is to describe the 30-year experience (1989–2019) in the surgical treatment of patients with gastric stump neoplasia at the Hospital de Clínicas, Universidade Estadual de Campinas.

## METHODS

Patients who underwent surgery for gastric stump cancer between 1989 and 2019 were included. Sex, age group, interval between the first surgery and diagnosis of gastric stump cancer, location of the ulcer that prompted gastrectomy, type of reconstruction, tumor resectability, surgery performed, reconstruction of the digestive tract, associated surgical procedures, postoperative complications using the Clavien-Dindo classification^4^, disease staging, and survival were analyzed.

The sample profile for categorical variables was described using absolute frequency (n) and percentage (%) values. Descriptive measures (mean, standard deviation) were obtained to describe quantitative variables. Kaplan-Meier curves were constructed to assess overall survival and survival stratified by factors. The significance level adopted for the study was 5%. The research was approved by the Institution’s Ethics Committee (CAEE No. 46462621.0.0000.5404).

## RESULTS

In total, 51 patients with a mean age of 66.9 years (standard deviation: 9.6 years) were evaluated, of whom 43 (83.3%) were men. The interval between the initial gastrectomy and surgery for the treatment of gastric stump cancer was 34.7 years (standard deviation: 10.9 years). Among the clinical comorbidities in this group of patients, smoking (35 patients; 76.1%) and alcoholism (24 patients; 53.3%) stood out.

All patients in this series had undergone Billroth II reconstruction at the time of diagnosis of gastric stump cancer. The indication for the initial gastrectomy was due to gastric ulcer in 28 patients (56%) or duodenal ulcer in 22 (44%). In one patient, it was not possible to obtain this information.

Preoperative staging using upper digestive endoscopy and computed tomography showed 12 (23.5%) patients with early tumors, 23 (45.1%) patients with advanced tumors, and 16 (31.4%) with metastatic tumors.

The indicated surgical treatment was total gastrectomy (35 patients – 68.6%) and subtotal gastrectomy (6 patients – 11.8%). Post-gastrectomy reconstructions were always performed with a Roux-en-Y anastomosis. A jejunostomy was temporarily left in patients undergoing total gastrectomy for postoperative nutritional support^21^. The mean number of resected lymph nodes was 18.3 (standard deviation: 13.1), and the mean number of lymph nodes affected by the neoplasia was 4 (standard deviation: 5.7). In 10 patients (19.6%), the intraoperative findings showed an unresectable lesion, and only jejunostomy was indicated for nutritional support, followed by chemotherapy. Table 1 shows the associated resections performed.

**Table 1:** Multi-visceral resections (13 patients – 25.4%).

	n (%)
Splenectomies	13 (25.4)
Cholecystectomies	11 (21.5)
Body-tail pancreatectomy	4 (7.8)
Partial colectomies	3 (5.8)
Enterectomies	3 (5.8)
Hepatectomies (II/III segments)	2 (3.9)
Left nephrectomy	1 (1.0)

Excluding patients with early tumors, the remaining gastrectomized patients underwent adjuvant chemotherapy, with the CROSS^12^ regimen being the most frequently used, considering the tolerance of each case.

Postoperative complications according to the Clavien–Dindo classification^4^ considered severe (Clavien-Dindo III or higher) were observed in 25 (49%) patients, the most frequent being pneumonia in 3 (12%) patients. Esophagojejunal anastomotic fistula occurred in 2 patients (5.7%).

The anatomopathological study of the surgical specimens showed a predominance of poorly differentiated adenocarcinomas in 21 cases (45.7%) and moderately differentiated in 17 of the cases (37%). Most patients had advanced tumors (23 cases – 48.9%), while 12 patients were diagnosed with early adenocarcinomas (25.5%). Hospital mortality was 4 patients (7.8%).

The mean follow-up time in this series was 34.2 months (standard deviation: 47.6 months). During the postoperative follow-up period, 6 patients were alive (11.7%), under outpatient follow-up.

**Figure 1:**
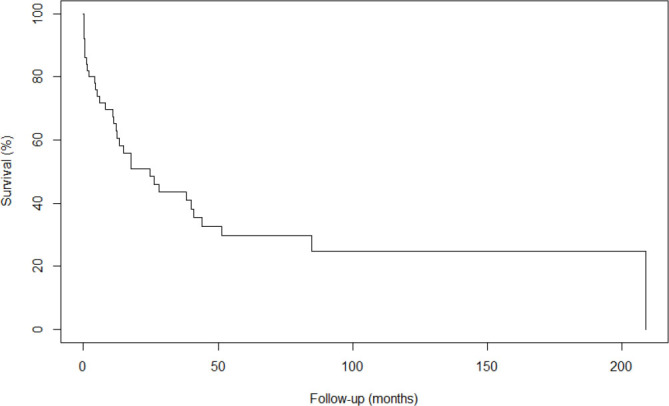
Kaplan-Meier curve for overall survival of patients operated on for gastric stump neoplasia.

The overall survival rate at 3 years was 43.6%, and the survival rate at 5 years was 29.7%. The Kaplan-Meier curve for overall survival in months is as follows (Figure 1).

**Figure 2:**
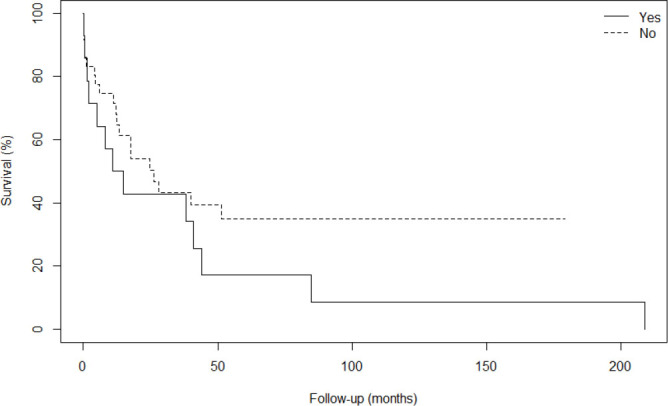
Kaplan-Meier curve of overall survival of patients operated on for gastric stump neoplasia, who underwent total or subtotal gastrectomy without resection of other organs (No), compared with patients who underwent multivisceral resections (Yes).

Figure 2 shows the Kaplan–Meier curve comparing the survival time of patients who underwent total or subtotal gastrectomy (associated or not with cholecystectomies) (28 patients – 54.9%) and those who underwent multi-visceral resections, as described in Table 1 (13 patients – 25.4%).

## DISCUSSION

Gastric stump neoplasia is an uncommon condition characterized by the development of cancer in the remaining stump of the stomach after partial gastrectomy^14^. This condition predominantly affects men, with a ratio of 3:1 in relation to women, as indicated by a meta-analysis by Mak et al.^8^. Studies that investigated prognostic differences between sexes did not find statistically significant differences^19^.

The risk of developing gastric stump neoplasia increases considerably after 15 years from the first gastric resection, as evidenced by several authors. This increased risk can be attributed to several factors, including changes in the remaining gastric mucosa over time and possible cumulative effects of exposure to environmental and genetic risk factors^1^. This finding is essential to guide prevention strategies and screening programs, especially in high-risk populations, such as elderly men who have undergone partial gastrectomy^14,15^.

Advanced age is a risk factor present in gastric stump neoplasia and primary gastric cancer, with diagnosis usually occurring after the sixth decade of life^12,14^. This results in lower rates of curative resection and a less favorable prognosis for affected patients. Epidemiological studies vary in the prevalence of gastric stump neoplasia, with some reporting an incidence as low as 0.8% and others as high as 12.9%, depending on the population studied and the detection method^8^.

In the present study of 51 patients, there was a predominance of male patients, with an advanced mean age, and the interval between the first gastric resection and surgery for the treatment of gastric stump neoplasia was considerably long, with a mean of 34.7 years, findings consistent with the literature.

Mason et al.^9^ and Offerhaus et al.^11^ suggested that pancreaticoduodenal reflux and postoperative hypochlorhydria may contribute to carcinogenesis due to the production of N-nitroso carcinogens in the gastric stump. Andreollo et al. observed the development of adenocarcinoma in animal models submitted to gastrojejunal anastomosis under the effect of duodenopancreatic reflux, under stimulation of carcinogens^1,9,11^. In the early 1990s, studies reinforced the idea that atrophy of the gastric mucosa, secondary to gastrectomy, creates a favorable precancerous environment, facilitating subsequent mutations that lead to carcinoma. The choice of gastric reconstruction type, such as Billroth I versus Billroth II, has been the subject of debate. Although studies such as those by Caygill et al.^3^ have associated a higher risk of gastric stump neoplasia with Billroth II reconstruction, meta-analyses such as that by Tersmette et al. have not found statistically significant differences^3,5,20^. However, it is currently recommended that all patients undergoing partial gastrectomy receive Roux-en-Y reconstruction to minimize duodenogastric reflux and thus reduce the risk of gastric stump cancer^7,14,15^.

Ramos et al. analyzed 54 patients with gastric stump neoplasias between 2009 and 2019 and found similar results, but highlighted a higher risk of esophagojejunal anastomotic fistula in the postoperative period^14^. Likewise, complications such as fistulas and pneumonia were the most frequent serious complications in the present study.

The recommendation is that patients who underwent Billroth I and Billroth II gastrectomies undergo periodic endoscopic control. Digestive endoscopy followed by biopsies is the most indicated examination for the diagnosis of primary gastric neoplasia and also for gastric stump neoplasia^12,15^.

Finally, this study showed that most diagnoses of gastric stump cancers were already performed as advanced tumors, which resulted in limited survival rates at 3 and 5 years. However, the authors of this study believe that efforts should be made to achieve an early diagnosis of adenocarcinoma in patients with gastric remnants, with periodic digestive endoscopies being recommended after 15 years of the previous gastrectomy.

## CONCLUSIONS

The treatment of gastric stump neoplasia is still challenging and difficult, and personalized follow-up strategies should be focused on high-risk patients, offering opportunities for early intervention, better clinical outcomes, and long-term survival.
